# Hematocolpos Presenting as Acute Urinary Retention: When Gynecologic Pathology Mimics Urologic Disease

**DOI:** 10.7759/cureus.95827

**Published:** 2025-10-31

**Authors:** Nasir Mahmood, Abdul Rehman, Syeda Zoya Chishti, Fazeelah Bibi

**Affiliations:** 1 Emergency Medicine, St. Luke's General Hospital, Kilkenny, IRL; 2 Obstetrics and Gynaecology, Pakistan Institute of Medical Sciences Hospital, Islamabad, PAK

**Keywords:** adolescent, hematocolpos, imperforate hymen, primary amenorrhea, urinary retention

## Abstract

An imperforate hymen is a rare congenital anomaly that typically presents at puberty with cyclic lower abdominal pain, primary amenorrhea, and, in severe cases, acute urinary retention secondary to mass effect from hematocolpos or hematometra. We report the case of a 13-year-old girl who presented with a 24-hour history of urinary retention, preceded by two months of dull lower abdominal pain, pressure, and dysuria. Pelvic ultrasound demonstrated a large, well-circumscribed cystic mass posterior to the bladder with internal echoes and endometrial thickening. Magnetic resonance imaging (MRI) confirmed a markedly distended vagina with blood signal characteristics consistent with hematocolpos. The patient underwent a cruciate hymenectomy under general anesthesia with evacuation of approximately 600 mL of retained blood.

In adolescent girls presenting with primary amenorrhea, cyclic pelvic pain, and urinary complaints, an imperforate hymen with resultant hematocolpos should be considered in the differential diagnosis. Prompt diagnosis and surgical management provide rapid symptom relief and prevent urinary and reproductive complications.

## Introduction

An imperforate hymen is a rare congenital anomaly and the most frequent cause of outflow obstruction in the female genital tract, with an estimated incidence of approximately 1 in 1,000 female births [1]. Most affected individuals remain asymptomatic until puberty, when menstrual blood accumulates behind the intact hymenal membrane, leading to hematocolpos or hematometra [2].

The buildup of retained blood can cause cyclic lower abdominal or pelvic pain, abdominal distension, and a palpable pelvic mass. In some cases, the resulting mass effect can produce urinary symptoms such as frequency, retention, or even hydronephrosis due to compression of the urethra or ureters [1]. Although urinary retention is uncommon in females, it occurs in up to 20% of cases of imperforate hymen and may rarely lead to acute kidney injury [2,3].

Early recognition through careful clinical assessment and appropriate imaging is essential to prevent complications and preserve future fertility and renal function [3]. We present the case of a 13-year-old girl with hematocolpos secondary to an imperforate hymen who presented with acute urinary retention.

## Case presentation

A 13-year-old girl with a past medical history of hay fever and no prior surgeries presented to the emergency department with severe lower abdominal pain and an inability to pass urine for 24 hours. This was preceded by two months of dull, persistent lower abdominal pain, pelvic pressure, and dysuria. On further questioning, she reported experiencing cyclic lower abdominal cramps for approximately one year. She had not yet attained menarche.

On general examination, the patient was afebrile but tachycardic, with a heart rate of 135 beats per minute. Blood pressure and oxygen saturation were within normal limits. Abdominal examination revealed suprapubic distension and tenderness, with a firm, mobile, renitent mass extending up to the umbilicus, consistent with a distended urinary bladder. A perineal examination demonstrated a bulging, imperforate hymenal membrane without an opening. Secondary sexual characteristics were consistent with Tanner stage V, indicating full pubertal development [4].

Bedside pelvic ultrasonography revealed a markedly distended urinary bladder and a well-circumscribed cystic structure posterior to it, without hydronephrosis. A urinary catheter was inserted, draining 700 mL of clear urine, which relieved her discomfort. Urine dipstick testing was negative for leukocytes and nitrites, and a pregnancy test was negative.

**Figure 1:**
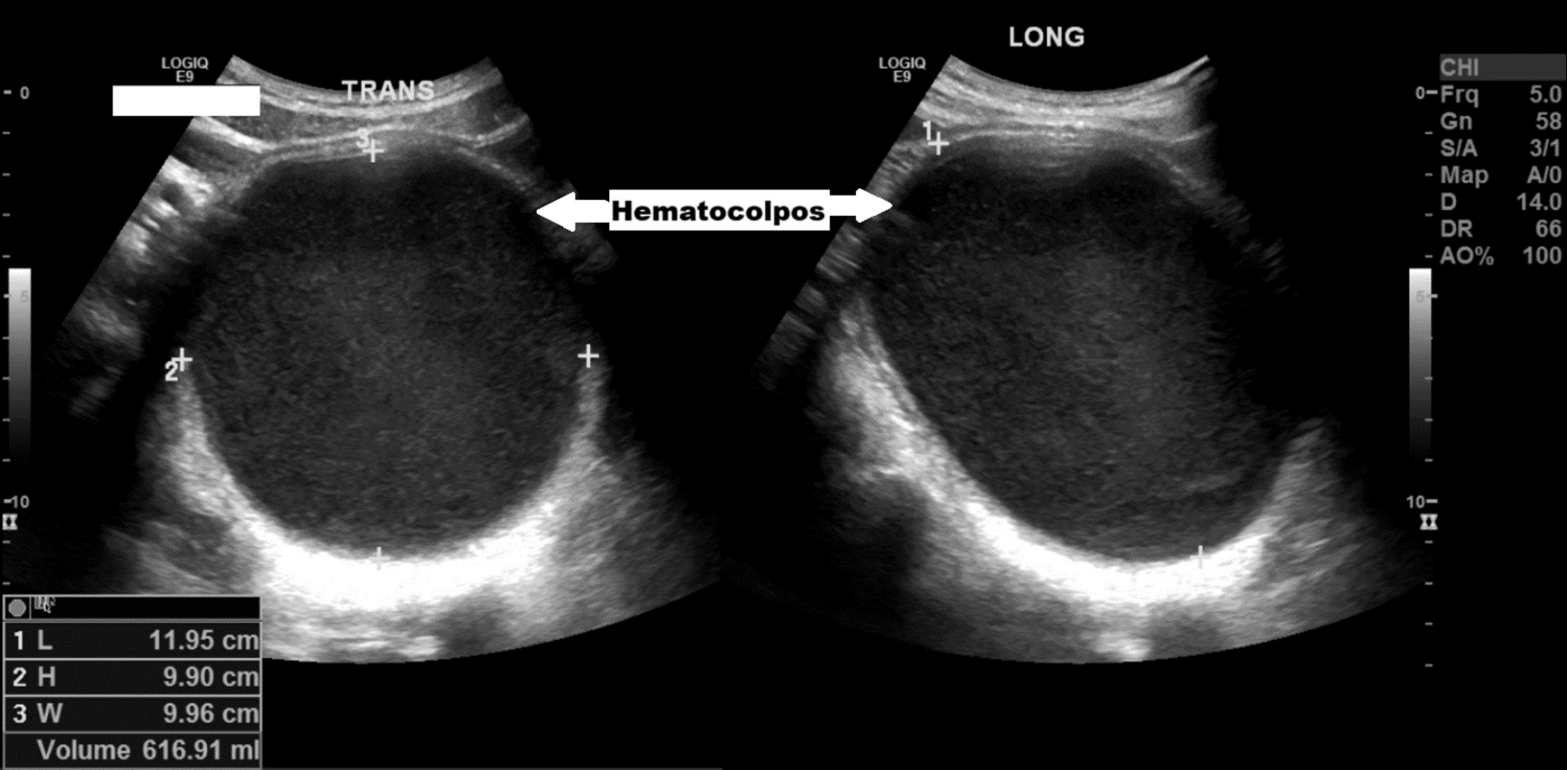
Pelvic ultrasound showing a well-defined cystic lesion with internal echoes, measuring 9.90 × 9.96 × 11.95 cm (estimated volume approximately 617 mL), arising from the cervical region, consistent with hematocolpos.

Formal pelvic ultrasound, performed by the radiologist, showed a normal uterus with endometrial thickening of 12 mm and a well-defined cystic lesion with internal echoes measuring 9.90 × 9.96 × 11.95 cm (volume approximately 617 mL), arising from the cervical region and displacing the bladder anteriorly (Figure 1). Both ovaries and kidneys appeared normal, and no free fluid was noted in the pelvis.

**Figure 2:**
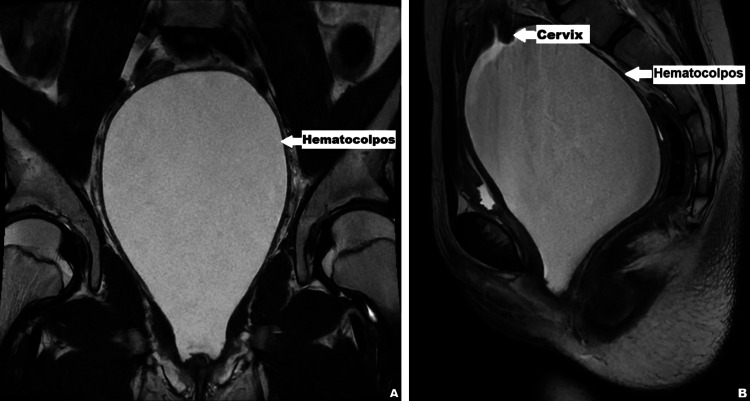
Coronal (A) and sagittal (B) MRI images demonstrating a distended vaginal cavity consistent with hematocolpos. The hyperintense signal on T2-weighted imaging indicates blood collection within the vagina, secondary to outflow tract obstruction. MRI: magnetic resonance imaging.

Given the suspicion of an obstructive genital anomaly, an urgent pelvic magnetic resonance imaging (MRI) scan was obtained. MRI demonstrated a markedly dilated vagina and uterus containing high T1 and intermediate-to-low T2 signal intensity material, consistent with blood products, measuring approximately 10 × 10 × 13 cm (Figure 2). These findings confirmed the diagnosis of hematocolpos and hematometra secondary to an imperforate hymen. No Müllerian duct anomalies were identified.

Under general anesthesia, the patient underwent a cruciate hymenectomy. A cross-shaped incision was made over the hymenal membrane, and approximately 600 mL of altered menstrual blood was drained. Care was taken to avoid excessive compression of the uterus to prevent retrograde reflux of blood into the fallopian tubes and peritoneal cavity, thereby reducing the risk of endometriosis or tubal injury. The edges of the hymenal incision were trimmed and sutured to the vaginal vestibule with absorbable sutures to prevent re-adhesion. Postoperatively, prophylactic antibiotics and analgesics were prescribed.

The postoperative course was uneventful. The patient was discharged on the second postoperative day and advised to attend outpatient follow-up after six weeks. At review, she was symptom-free, voiding normally, and reported no recurrence of abdominal pain or urinary difficulties. Examination revealed a well-healed hymenal opening with no signs of re-adhesion or other complications.

## Discussion

This case represents a classic yet frequently underrecognized presentation of imperforate hymen complicated by significant hematocolpos resulting in acute urinary retention. Although rare, an imperforate hymen remains the most common obstructive anomaly of the female genital tract [1]. It results from the failure of the inferior portion of the vaginal plate to canalize during embryologic development [1].

Symptoms typically manifest at puberty when the obstruction prevents menstrual blood from exiting the vagina, leading to hematocolpos, hematometra, or hematosalpinx [2,3,5]. The accumulation of retained blood causes progressive pelvic distension and can produce cyclic lower abdominal pain, urinary frequency, or acute urinary retention due to anterior compression of the urethra and bladder neck. Severe cases may progress to hydronephrosis or acute kidney injury. Previous reports indicate that approximately 20% of imperforate hymen cases present with urinary retention, and about 2% develop renal involvement [3].

Physical examination remains central to diagnosis. Assessment of pubertal development using the Tanner scale is essential to determine the stage of sexual maturity and to contextualize clinical findings [4,6]. A bulging, bluish hymenal membrane at the vaginal introitus is considered pathognomonic and should be specifically looked for in adolescent girls presenting with primary amenorrhea, cyclic pelvic pain, or unexplained urinary symptoms [7,8]. A simple genital inspection can often establish the diagnosis and prevent unnecessary delays or invasive procedures [7,8].

Ultrasound is typically the first-line bedside imaging modality. It demonstrates a midline cystic pelvic mass posterior to the bladder, often containing internal low-level echoes representing menstrual blood. However, large collections may obscure anatomical detail, making it difficult to exclude associated anomalies [9-11]. In such cases, magnetic resonance imaging (MRI) provides superior soft-tissue resolution and is considered the gold standard for confirming the diagnosis and assessing uterine and vaginal anatomy, as well as identifying any Müllerian duct anomalies [12-14].

The mainstay of treatment is surgical creation of a patent outflow tract, typically via hymenectomy or hymenotomy. The cruciate (cross-shaped) incision with excision of redundant hymenal tissue and eversion or suturing of the edges to the vestibule is the preferred approach, as it minimizes the risk of re-adhesion [15]. Other incision patterns, that is, vertical, T-shaped, or circular, may be chosen based on surgeon preference and anatomical considerations. During the procedure, gentle evacuation of retained blood is crucial; excessive compression of the uterus should be avoided to prevent retrograde flow into the fallopian tubes, which could predispose to endometriosis, iatrogenic pelvic infection, or pelvic adhesions [16,17].

Postoperative management includes antibiotic prophylaxis and, in some cases, the application of topical estrogen to reduce the risk of hymenal reclosure, particularly in younger or prepubertal patients. Recurrence is uncommon, but follow-up is recommended to ensure proper healing and assess for any residual or associated anomalies [15]. In patients with distorted pelvic anatomy or incomplete visualization during the initial assessment, a postoperative MRI may be warranted to exclude coexisting Müllerian anomalies such as a transverse vaginal septum or cervical agenesis [17].

This case aligns with previously reported instances describing urinary retention secondary to an imperforate hymen [2,3,5-9]. Similar cases in the literature have documented adolescent patients presenting with acute urinary retention who, upon catheterization, passed large volumes of urine followed by evacuation of significant quantities of retained menstrual blood during hymenotomy [2,3,5-11]. These findings emphasize the importance of considering gynecologic causes in female patients presenting with urinary retention, despite the rarity of this symptom in females.

Several key clinical lessons can be drawn from this case. First, an imperforate hymen should always be considered in adolescent girls presenting with primary amenorrhea, cyclic pelvic pain, or urinary symptoms. Second, a careful external genital examination is a simple yet essential diagnostic step that can lead to an immediate diagnosis and prevent unnecessary delays in management. Third, imaging, particularly MRI, plays a valuable role in confirming the diagnosis, guiding surgical planning, and identifying any associated Müllerian anomalies. Finally, timely surgical intervention offers rapid symptom relief, prevents complications, and preserves reproductive function.

While the clinical outcome in this case was favorable, certain limitations should be acknowledged. The absence of long-term follow-up data regarding menstrual regularity and fertility outcomes limits the assessment of the patient’s reproductive prognosis. Additionally, the inclusion of intraoperative and postoperative imaging could have further strengthened the report by providing visual confirmation of the diagnosis and treatment outcome.

In conclusion, an imperforate hymen, though uncommon, should remain an important consideration in adolescent girls presenting with primary amenorrhea and pelvic or urinary complaints. Prompt recognition through careful examination and appropriate imaging, followed by timely surgical management, is crucial for symptom resolution, prevention of urological complications, and preservation of future fertility.

## Conclusions

An imperforate hymen complicated by hematocolpos should be included in the differential diagnosis of adolescent females presenting with primary amenorrhea, cyclic lower abdominal pain, and urinary retention. A high index of suspicion, careful history-taking, external genital and perineal examination, and appropriate imaging are essential for accurate diagnosis. Prompt surgical intervention provides rapid relief of symptoms, restores normal menstrual flow, and preserves reproductive potential.

An imperforate hymen, though the most common obstructive anomaly of the female genital tract, rarely presents with acute urinary retention. This case highlights how a concealed developmental anomaly can masquerade as a urological emergency, delaying recognition, and emphasizes the critical role of early clinical suspicion and multidisciplinary management in ensuring optimal outcomes for patients with this correctable congenital anomaly. A simple genital and perineal inspection, often overlooked in adolescent girls with abdominal or voiding complaints, can expedite diagnosis and prevent prolonged morbidity. The rarity of urinary retention as the sentinel event underscores the need for a multidisciplinary lens (pediatric, urologic, and gynecologic) when evaluating unexplained voiding dysfunction in peripubertal females. Early recognition and timely hymenectomy not only resolve symptoms but also safeguard reproductive potential, making vigilance essential in seemingly routine presentations.
